# Adrenal Cyst Presenting as Hepatic Hydatid Cyst

**DOI:** 10.1155/2013/150457

**Published:** 2013-04-02

**Authors:** Abdulla Darwish, Veena Nagaraj, Mohmmed B. Mustafa, Ahmed Al Ansari

**Affiliations:** ^1^Pathology Department, Bahrain Defense Force Hospital, P.O. Box 28347, Riffa, Bahrain; ^2^Department of General Surgery, Bahrain Defense Force Hospital, P.O. Box 28347, Riffa, Bahrain

## Abstract

*Introduction*. Although adrenal cysts are uncommon, the incidence rate is increasing with the advances in radiological technologies. The incidental detection of adrenal cysts nowadays has become more frequent as a result of the increase usage of high quality imaging modalities. Adrenal cysts originate from the adrenal gland and can be classified into either true or pseudocyst. *Presentation of Case*. In this report, we described an adrenal cyst of endothelial type, in a 30-year-old lady who was mistakenly diagnosed to have a hydatid cyst both clinically and by imaging. *Discussion*. Although adrenal cysts are uncommon, the incidence rate is increasing with the frequent use of various high quality radiological technologies. Adrenal cyst should be considered in the differential diagnosis when dealing with upper abdominal cysts. The size of the adrenal cyst can vary from a few millimeters up to 50 cm in diameter. Most of the adrenal cysts are unilateral, while 8%–15% of those cysts do present bilaterally. The majority of cases are diagnosed between the 3rd and 5th decades. *Conclusion*. Although most of the adrenal cysts are benign in nature, surgical excision is advisable especially when the cysts are greater than 5 cm in diameter and in the case of suspecting malignancy.

## 1. Introduction

Most of the adrenal cysts are asymptomatic and heterogeneous in nature [1, 2]. Big cysts can present incidentally, or due to the mass effect compressing on adjacent structures causing abdominal or flank pain [3]. Today, with the improvement in imaging modalities and increase in their usage, adrenal cysts are being detected more frequently as incidental lesions [3].

 In 1989, Medeiros et al. stated that around 300 cases were discovered to that date [4]. In a literature review in 1999, Neri and Nance described that 34% of all adrenal cysts are discovered incidentally, and 39% present with abdominal pain or due to mass compression effect [5]. 

In 2010, Wedmid and Palese documented that more than 600 adrenal cystic lesions were reported in the literature [3, 6]. Adrenal cysts can present unilaterally or bilaterally. Although that the majority of the adrenal cysts are unilateral, 8%–15% are bilateral without side predominance [7]. We present a true adrenal cyst of endothelial type closely attached to the hilum of the liver, which was misdiagnosed clinically and radiologically as a Hydatid cyst.

## 2. Case Report

**Figure 1:**
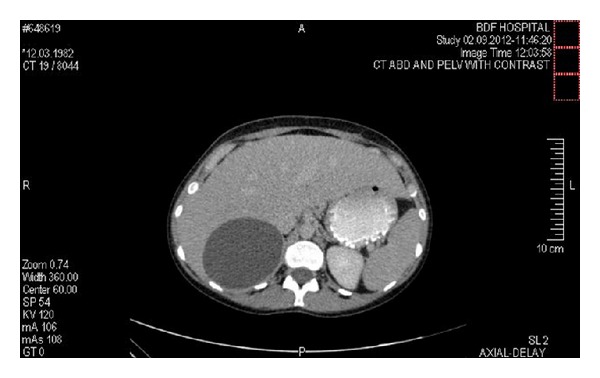
CT abdomen and pelvis showing the cyst.

A 30-year-old female with a history of bronchial asthma and pregnant at 14 weeks of gestation presented with hyperemesis gravidarum to one of the private hospitals in the city. Abdominal ultrasound was performed showing a large cyst in the liver measuring 12 × 7 cm in size, which was diagnosed as a hydatid liver cyst. On general examination, signs of dehydration were detected. Abdominal examination revealed mild, diffuse upper abdominal tenderness. She was given symptomatic treatment and referred to the surgical clinic for followup, which she failed to attend. After delivery and during the routine follow-up visit, abdominal ultrasound was done showing evidence of a bilocular cyst with internal septa measuring 15 × 7 cm situated in the right lobe of the liver with an extension to the undersurface. CT scan of the abdomen and pelvis revealed a septated cyst arising from the right lobe of the liver (Figure 1). A calcified septation within the cyst and some degree of calcification of the superior aspect of the cyst were detected. The left adrenal gland, spleen, and both kidneys were all normal. However, the right adrenal gland could not be delineated. All other investigations including chest X-ray, renal function tests, liver function tests, other biochemical tests, electrolytes, hematocrit, and thyroid function tests were normal. Urine culture and sensitivity and viral serology were all normal. Antibodies for a hydatid cyst were negative (160, reference range < 360). Due to the large size of the cyst, surgical intervention was considered, laparotomy was performed, and the cyst was excised. 

**Figure 2:**
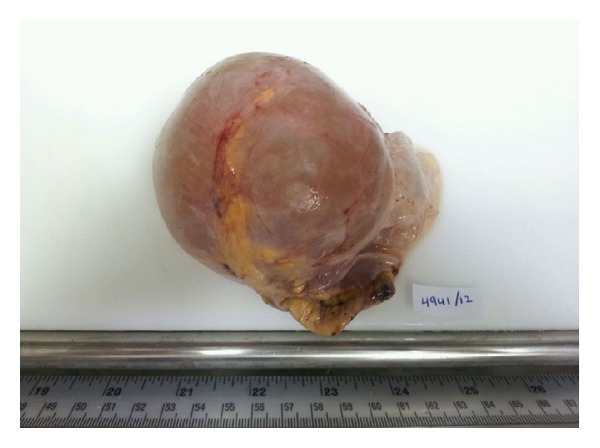
Macroscopic appearance of the specimen showing tense glistening grey brown cyst filled with clear fluid. Note the residual adrenal tissue attached to the external surface.

**Figure 3:**
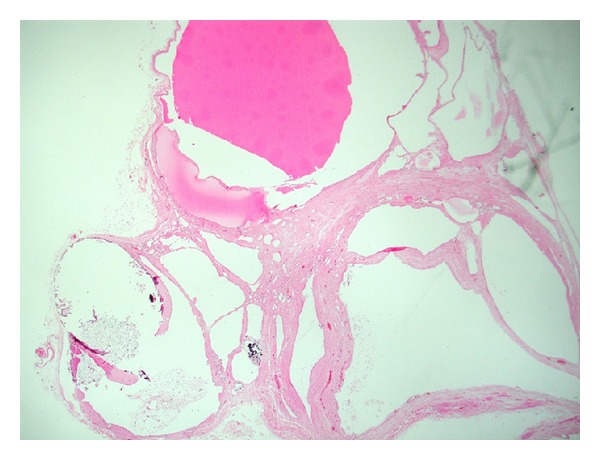
low power view of the adrenal cyst composed of multilocular spaces filled with eosinophilic material. Note the presence of calcification at the lower left side of the photo (H&E stained slide).

**Figure 4:**
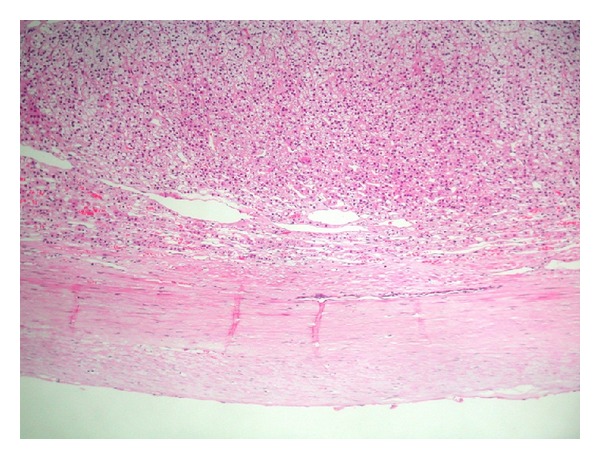
This photo shows a fibrocollagenous cyst wall with attached adrenal tissue (H&E stained slides).

**Figure 5:**
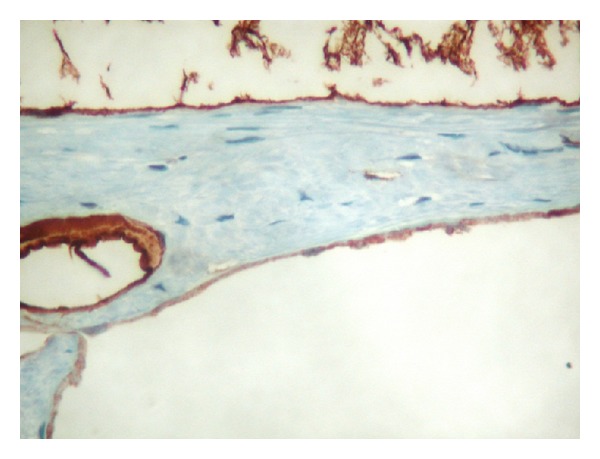
showing cystic cell lining positive for factor VIII confirming endothelial cell origin (immunohistochemistry stained slide).

 Macroscopic examination of the specimen showed a 12 × 9 × 7 cm partially collapsed multilocular brownish glistening cyst, weighing 210 grams and containing serous fluid (Figure 2). The outer surface showed a 4 × 2 × 0.5 cm yellowish fatty adrenal tissue attached to the cyst. The average wall thickness of the cyst was 0.2 cm; the inner lining was largely smooth with focal roughened papilloid areas. Microscopically, the multilocular cyst showed a focally calcified fibrotic cyst wall lined by low cuboidal to flattened epithelium which showed strong positivity for factor-8 immunohistochemistry (Figures 3, 4, and 5). Congested blood vessels and clusters of lipidized adrenal cortical cells were also seen in the stroma. The included adrenal gland was within normal limits. No pleomorphism or mitosis was seen. The overall appearance was consistent with a benign adrenal cyst of endothelial type.

## 3. Discussion

 Since 1670s, when the first adrenal cyst was described, there has been an evolution in the various radiological modalities, which has contributed to the incidental diagnosis of adrenal cysts [3]. Although adrenal cysts are uncommon, the incidence rate is increasing with the frequent use of high quality radiological imaging techniques. The most common type of adrenal cysts reported in the literature is the nonfunctional cyst. However, adrenal cysts can present in different forms; they can be small or large, functional or nonfunctional, true or pseudocysts, and benign or malignant [2, 8]. On pathological examination, the majority of adrenal cysts are categorized as either pseudocysts or true cysts. The incidence rate of adrenal cysts varies from one type to another. In the literature, pseudocysts account for 60% of the cases and can be classified into hemorrhagic cysts, neoplastic, and parasitic cysts. No epithelial lining is found in this type of adrenal cysts [2, 3, 8]. True adrenal cysts account for 40% of the cases and can present as endothelial cysts and epithelial cysts and rarely as vascular cysts or cystic lymphangiomas [3, 8]. 

 Most of the adrenal cysts are asymptomatic and can be diagnosed incidentally, more commonly situated in the right side, and can be detected more frequently in female patients [3, 8]. The size of the adrenal cysts can vary from few millimeters up to 50 cm in diameter [9]. Most of the adrenal cysts are unilateral, with 8%–15% that can present bilaterally [7]. The majority of cases are diagnosed between the 3rd and 5th decades [10, 11]. An adrenal cyst located in the left side can be confused for a pancreatic pseudocyst, while a right-sided adrenal cyst can be confused for a liver cyst [12], as presented in this case.

 Investigations can range from hormonal workup to radiological modalities. CT scan is the investigation of choice for the diagnosis of adrenal cysts. It can show the location of the cyst, reveal the density, and define the wall borders. MRI is superior in the differential diagnosis and in distinguishing between an adrenal lesion and a renal lesion [13]. Hormonal workup in the form of a 24-hour urinary metanephrine, serum potassium for three consecutive days, a low-dose dexamethasone suppression test, and aldosterone: renin ratio is necessary in all cases of adrenal cysts in order to diagnose functional disease that is not clinically detected [8].

 Adrenal cysts can be managed either conservatively or by surgical excision. Management is dependent on the functional status of the cyst, the probability of malignancy, and complications such as hemorrhage or infection of the cyst [1, 3, 4]. Excision or aspiration of the adrenal cyst is recommended if the patient is symptomatic and presenting with endocrine abnormalities, or with rapid expansion of the cyst, and when malignancy cannot be ruled out [14]. In addition, Bellantone et al. have recommended surgical resection of cysts that are greater than 5 cm due to the risk of hemorrhage and infection [7]. Surgical resection will decrease the incidence of future complications in the form of hemorrhage into the cyst, local pressure effect and infection of the cyst, and to exclude the possibility of malignancy [1]. 

## 4. Conclusion

 Adrenal cysts should be considered in the differential diagnosis when dealing with upper abdominal cysts. Although most adrenal Cysts are benign in nature, surgical excision is advisable especially when the cysts are larger than 5 cm in diameter, and there is a suspicion of malignancy.
